# Consensus on the Southeast Asian management of hypotension using vasopressors and adjunct modalities during cesarean section under spinal anesthesia

**DOI:** 10.1186/s44158-022-00084-1

**Published:** 2022-12-28

**Authors:** Grace Anne B. Herbosa, Nguyen Ngoc Tho, Angelina A. Gapay, Suraphong Lorsomradee, Cong Quyet Thang

**Affiliations:** 1 Department of Anesthesiology, University of the Philippines College of Medicine, Manila, Philippines; 2 Department of Anesthesiology and Intensive Care, Hanoi French Hospital, Hanoi, Vietnam; 3 Department of Anesthesiology, Divine Word Hospital, Tacloban, Philippines; 4 Department of Anesthesiology, Faculty of Medicine, Chiang Mai University Hospital, Chang Mai, Thailand; 5 Vietnam Society of Anesthesiologists, Head of Department of Anesthesiology and SCIU at HuuNghi Hospital, Hanoi, Vietnam

**Keywords:** Blood pressure measurement, Cesarean section, Ephedrine, Hypotension, Phenylephrine, Spinal anesthesia, Vasopressor

## Abstract

**Background and aims:**

This consensus statement presents a comprehensive and evidence-based set of guidelines that modify the general European or US guidelines for hypotension management with vasopressors during cesarean delivery. It is tailored to the Southeast Asian context in terms of local human and medical resources, health system capacity, and local values and preferences.

**Methods and results:**

These guidelines were prepared using a methodological approach. Two principal sources were used to obtain the evidence: scientific evidence and opinion-based evidence. A team of five anesthesia experts from Vietnam, the Philippines, and Thailand came together to define relevant clinical questions; search for literature-based evidence using the MEDLINE, Scopus, Google Scholar, and Cochrane libraries; evaluate existing guidelines; and contextualize recommendations for the Southeast Asian region. Furthermore, a survey was developed and distributed among 183 practitioners in the captioned countries to gather representative opinions of the medical community and identify best practices for the management of hypotension with vasopressors during cesarean section under spinal anesthesia.

**Conclusions:**

This consensus statement advocates proactive management of maternal hypotension during cesarean section after spinal anesthesia, which can be detrimental for both the mother and fetus, supports the choice of phenylephrine as a first-line vasopressor and offers a perspective on the use of prefilled syringes in the Southeast Asian region, where factors such as healthcare features, availability, patient safety, and cost should be considered.

## Key recommendations for best clinical practice

Using the best available evidence, the 2016 ACC and AHA Clinical Practice Guideline Recommendation Classification Systems [1] were used to evaluate each of the following elements (Table 1).

**Table 1:** Key recommendations for best clinical practices

Key recommendations	Strength of recommendations	References
Hypotension during SA needs prompt recognition and treatment, since it is frequent and has adverse effects on the mother and the fetus	High	[2, 3]
A systolic NIBP of < 80% of baseline is considered hypotension, and SBP must be maintained at > 90% of baseline	High	[2–4]
Vasopressor should be used to manage SA–associated hypotension	High	[4–6]
Phenylephrine should be used as the first-line of vasopressor treatment to maintain the desired SBP in the absence of bradycardia	High	[2, 5]
Phenylephrine can be given prophylactically to reduce the risk of hypotension and nausea and vomiting after SA	High	[2, 3, 7]
Phenylephrine may be administered as an infusion titrated at 25–50 µg/min after administration of SA, depending on blood pressure and heart rate with additional IV boluses if needed	High	[2, 3, 8]
Phenylephrine can be administered as a bolus of 50–100 µg on SA administration. For immediate management of hypotension, IV bolus of phenylephrine has a faster onset than an infusion	High	[2, 3]
Phenylephrine and ephedrine bolus should be administered using prefilled syringe since it prevents medication errors, creates less waste, improves patient safety, and allows long-term cost savings	High	[2, 9–11]
Intermittent IV boluses of 5–15 mg ephedrine must be administered in the presence of bradycardia and hypotension, but the cumulative dose before delivery should not exceed 15 mg to minimize fetal acidosis	High	[5, 12, 13]
Leg compression devices, manual left uterine displacement, wedge for left uterine displacement, and administration of 5HT3 antagonists (e.g., ondansetron) may be used as prespinal measures to prevent hypotension after SA	Medium	[5, 14–17]
Crystalloid co-loading should commence together with prophylactic administration of vasopressors	Medium	[2, 3]
Noradrenaline (norepinephrine) infusion (starting rate can be 0.1 µg /kg/min) or bolus (5–10 µg) may be used in limited-resource areas (using a central line or temporarily in large-bore peripheral line)	Low	[18–21]
An anticholinergic agent (glycopyrrolate or atropine) may be used for significant bradycardia with hypotension	Low	[2, 22]

## Background

Spinal anesthesia (SA) is generally used for elective cesarean section [23]. A common consequence of the sympathetic vasomotor block caused by SA for cesarean section is hypotension, which occurs in up to 80–90% of cases, depending on the definition [24, 25]. SA often leads to a drop in blood pressure and bradycardia by inducing a sympathovagal imbalance toward parasympathetic tone [26]. Cardiac output studies have shown that SA has a biphasic effect, in which cardiac output initially increases following a reduction in afterload from arterial vasodilatation and subsequently decreases due to a reduced preload [2]. Hypotension affects both the mother and fetus, including maternal nausea, vomiting, dizziness, rare loss of consciousness, cardiac arrest, and death [27]; and fetal symptoms, including low Apgar scores and low umbilical arterial pH (umbilical acidosis), have been shown to be associated with the duration and severity of hypotension [28]. Hypotension may be related to maternal and neonatal morbidity. Thus, several methods have been investigated alone or in combination for its prevention and treatment [2]. Left uterine displacement has been used to reduce the effects of aortocaval compression [14]. Simply elevating the legs is not sufficient to reduce the incidence of hypotension [15]. Fluid preloading is often performed with varying results [16, 17]. However, fluid co-loading is universally accepted.

Nonpharmacological techniques have shown uncertain efficacy in effectively treating hypotension, and a vasopressor is often required during SA for cesarean section. Several factors must be considered in choosing an appropriate vasopressor, such as its efficacy in maintaining blood pressure, maternal non-cardiovascular effects, ease of use, direct and indirect fetal effects, availability, and cost [2].

The vasopressors commonly used to manage hypotension during SA are ephedrine and phenylephrine [2]. The use of ephedrine for obstetric patients is supported by evidence from animal studies, which have shown better maintenance of uteroplacental blood flow when ephedrine is used to increase maternal blood pressure [29]. However, ephedrine has several disadvantages, including a slow onset and a relatively long duration of action, making accurate titration of blood pressure potentially difficult [30]. Clinical studies have shown that ephedrine is associated with a dose-dependent tendency to lower fetal pH and base excess [31].

Phenylephrine is a potent direct-acting alpha-adrenergic agonist. Relatively high doses of phenylephrine may be required in pregnancy because of the often reduced pressor response to endogenous and exogenous vasoconstrictors [32]. However, fetal acidosis following the use of phenylephrine to maintain maternal blood pressure and prevent symptoms has not been demonstrated [33].

This evidence-based consensus statement aims to translate findings from research into recommendations for clinical practice that, when implemented, can improve outcomes.

## Methods

**Fig. 1:**
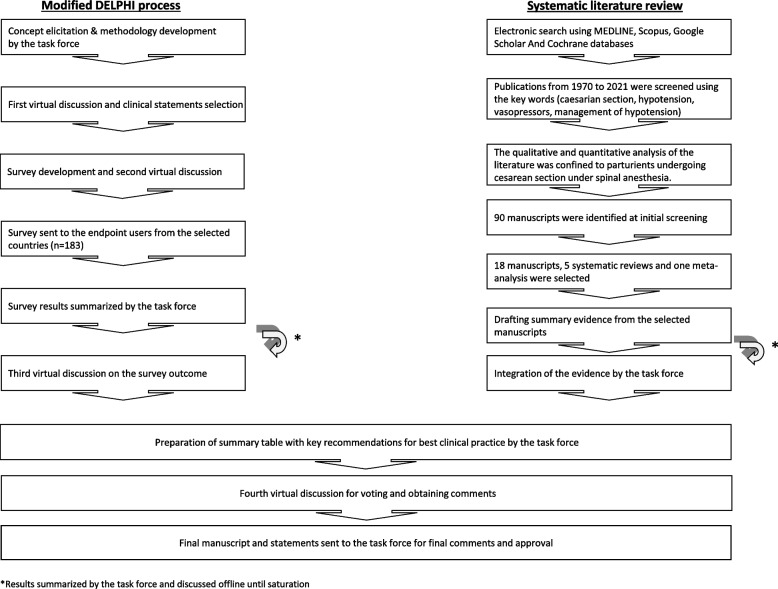
Flow diagram of the consensus steps, including a systematic literature review and modified Delphi process

This manuscript describes the several stages used to reach a consensus among selected experts in anesthesia after a careful review of existing evidence. A flow diagram of the consensus procedures, including the systematic literature review and the modified Delphi process, is shown in Fig. 1.

The whole process included four virtual meetings, 3 months of offline work, and two rounds of votes.

### Literature review

This study conforms to the preferred reporting points for systematic reviews and meta-analyses [34]. The MEDLINE, Scopus, Google Scholar, and Cochrane libraries were explored for the systematic literature review. A combination of subject headings and free-text terms was used. The search was limited to publications from 1970 to 2021. The quantitative and qualitative analysis of the literature were confined to parturients undergoing cesarean section under SA. We included the following interventions for hypotension: vasopressors (ephedrine, phenylephrine, norepinephrine, epinephrine), nonpharmacologic interventions (left lateral tilt, fluid co-loading, compression stockings, position changes), and pharmacological preventive strategies (local anesthetic dosage). Outcomes included maternal outcomes (presence of hypotension, degree of hypotension, duration of hypotension, nausea, and vomiting) and fetal outcomes (presence of acidosis, asphyxia, and death). During the initial search, 90 titles could be identified. After eliminating duplicates and irrelevant studies, the remaining 27 titles were reviewed, resulting in 18 titles being selected for analysis. In addition, five systematic reviews and one meta-analysis were considered.

### Modified Delphi process

A team of five anesthesiology experts from the Philippines, Vietnam, and Thailand were selected, based on their expertise assessed using a pedigree analysis, to define the relevant clinical questions, search for literature-based evidence, evaluate the available guidelines, and contextualize the recommendations for Southeast Asian (SEA) conditions. Furthermore, they compared the adapted guidelines with real-world practice through a survey shared with a representative panel of local practitioners to collect opinions, identify conflicting views, and determine best practices for the management of hypotension with vasopressors during cesarean section under SA based on current knowledge, evidence, and available treatments.

A group of 183 experts was selected and invited to participate in a web-based survey via email describing the aims and procedures. The survey was conducted online using SurveyMonkey® between June 2022 and September 2022. It consisted of 18 statements designed to gather the opinions of the members from the selected countries. The results of the survey were assessed using the percentage of responses for each statement on a Likert scale, which included “Strongly Agree”, “Agree”, “Disagree”, and “Strongly Disagree” response options. The consensus agreement was predefined as 80%. The analysis of the responses was performed by the panel members, and 17 consensus statements were developed and revised.

### Consensus recommendations

The literature review results combined with the modified Delphi survey contributed to the development of the consensus statement. Based on the best available evidence, the 2016 ACC and AHA Clinical Practice Guideline Recommendation Classification Systems [1] were used to evaluate each proposed recommendation. Each recommendation was rated in terms of its strength (weak or strong) and quality (high, moderate, low, very low) according to the Grading of Recommendations Assessment, Development and Evaluation (GRADE) methodology.

### Literature review

#### Definition of hypotension

There is no accepted definition of hypotension in the scientific literature. The incidence of hypotension varies depending on the chosen definition; even small alterations in the definition result in large differences in the incidence of hypotension [35]. The reported incidence of hypotension varies between 1.9% [36] and up to 80–90% [24, 25], a huge gap related to highly different definitions of hypotension between studies. In a 2010, systematic review of the literature that addressed the wide range of definitions of hypotension in studies of women undergoing SA for cesarean section, Klör et al. [35] found that the two most common definitions of hypotension were defined by a drop of < 80% from baseline or a combination of two criteria, i.e., SBP < 100 mmHg or a drop of < 80% from baseline. Other international studies on obstetric anesthesia favored a SBP < 20% baseline [8, 37], SBP < 90 mmHg [38], or a combined definition (SBP < 20% baseline or SBP < 90 mmHg) [39]. A survey of obstetric anesthetists in the UK found that the preferred absolute SBP thresholds were < 90 mmHg or < 100 mmHg [40]. In a recent (2018) international consensus statement on the treatment of spinal hypotension using vasopressors, the authors stated that mean arterial pressure (MAP) is a better indicator of organ perfusion, but should probably not be used because of the lack of supporting data [2]. They advise maintaining a SBP ≥ 90% of baseline until delivery of the newborn and reducing the number of SBP episodes to < 80% of baseline. Treatment for values that fall below this level should be rapid, preferably with vasopressors [2]. Importantly, they refer to a reduced pressure from baseline, because women in labor show higher arterial pressure. Absolute values may lead to the underdiagnosis of hypotension. The SBP threshold for pregnancy-induced hypertension or preeclampsia is > 140 mmHg [2].

#### Pathophysiology and consequences of hypotension

Nausea and vomiting occur significantly more frequently during SA for caesarean section than during non-obstetric surgery and are mainly caused by hypotension [41]. SA–induced hypotension is caused by arterial and venous vasodilatation that results from the sympathetic block, together with the paradoxical activation of cardioinhibitory receptors [2]. Acute hypotension activates vomiting centers by reducing cerebral perfusion and inducing transient brainstem ischemia [42]. This can also lead to transient cerebral hypoxia associated with a marked decrease in maternal cerebral blood volume, cerebral oxygen saturation, and oxygenation [43]. These effects are also consistent with the prevention of cerebral hypoxia and a reduction in the incidence of nausea by oxygen inhalation [44, 45]. Prolonged, severe maternal hypotension can cause dizziness and decreased consciousness [46], which is less likely to occur when a drop in blood pressure is immediately treated.

SA leads to an approximately 20% reduction in splanchnic blood flow, which is also notable in systemic hypotension [47]. Splanchnic hypoperfusion results in the release of emetogenic substances, such as serotonin from the digestive tract, another pathophysiological mechanism of nausea, and vomiting [42]. Finally, sympathetic blockade by SA leads to an unopposed vagal effect, resulting in gastrointestinal hyperactivity [48].

Bradycardia, defined as a reduction of > 20% heart rate at baseline after SA, must always be considered as a warning sign of an important hemodynamic compromise [49]. In serious bradycardia with hypotension, an anticholinergic (glycopyrrolate or atropine) may be required. However, current evidence is insufficient to recommend the routine use of glycopyrrolate for the prevention of hypotension [22].

Fetal perfusion depends on uteroplacental blood flow, which in turn depends on uterine perfusion. Newborns from mothers with SA–induced hypotension are significantly more acidotic [50]; however, the severity of hypotension plays a less important role than its duration [2].

#### Pharmacology of vasopressor agents

Vasopressors commonly used to prevent SA-induced hypotension include primarily the direct action of the selective α1-receptor agonist phenylephrine and the direct and indirect action of ephedrine [51]. Both epinephrine and norepinephrine are alternatives for the correction of hypotension and bradycardia that do not respond to ephedrine [52, 53].

#### Phenylephrine

At clinical doses, phenylephrine is a selective α1-receptor agonist and becomes a β-agonist at much higher doses [54]. It can also induce baroreceptor-mediated bradycardia when given at higher than required doses [2]. It counteracts SA-induced hypotension due to pronounced arterial vasoconstriction due to its actions as a α1-agonist [55]. An intravenous (IV) dose of phenylephrine shows an almost immediate onset of action and a duration of action of 5–10 min [56]. Phenylephrine shows a faster onset of action than ephedrine (40 vs. 90 s, respectively) [13]. A number of studies suggest that SA-induced hypotension is prevented by an intermittent bolus dose (ED95) of phenylephrine of at least 122–147 µg [39, 57] administered intravenously; however, a 40–100 µg bolus dose is still a common clinical practice [58]. Prophylactic infusions in the range of 25–100 µg/min have been recommended. A fixed dose of 50 µg/min can minimize the risk of a higher incidence of hypotension, reactive hypertension, bradycardia, and decreased cardiac output [8, 55, 58].

#### Ephedrine

Ephedrine has a weak direct α- and β-agonistic effect; however, its indirect effect is greater because of the release of norepinephrine from sympathetic neurons [54]. It increases blood pressure by stimulating the β1-receptor, resulting in increased heart rate and cardiac contractility, whereas the α-agonist effect leads to peripheral vasoconstriction [59, 60]. Ephedrine crosses the placenta to a greater extent because of high-lipid solubility and undergoes less fetal metabolism and redistribution. This leads to increased fetal concentrations of lactate, glucose, and catecholamines, resulting to fetal acidemia due to stimulation of fetal adrenergic receptors. Its overall effect on fetal oxygen supply and demand balance may favor phenylephrine over ephedrine [61]. As a rescue vasopressor, a 5–15 mg bolus administered intravenously is most often recommended for treating hypotension following SA. The drug has a delayed onset of action and a longer duration of action of approximately 60 min. The depletion of presynaptic norepinephrine stores also leads to tachyphylaxis [62]. Thus, IV boluses are preferable for continuous IV infusion [2]. To avoid fetal acidemia, ephedrine is a poor choice and a maximum dose of 15 mg ephedrine is recommended, if it is the only vasopressor available.

#### Norepinephrine

Norepinephrine (noradrenaline) is a potent α-adrenoceptor agonist and a weak β-adrenoceptor agonist. It is, therefore, an attractive option for maintaining maternal blood pressure with fewer adverse effects on heart rate and cardiac output. In addition, it shows the same efficacy as phenylephrine in preventing and treating SA-induced hypotension during cesarean delivery [63]. Blood pressure, stroke volume, and neonatal outcomes are similar for patients who receive norepinephrine or phenylephrine. Nonetheless, the incidence of maternal bradycardia is lower with norepinephrine [18]. Other studies have shown that norepinephrine may be an alternative to phenylephrine without adverse effects [19, 20]. However, data are still lacking, indicating that norepinephrine is a better alternative to phenylephrine regarding fetal acidemia. Also, the placental transfer is minimal compared to ephedrine.

#### Epinephrine

Relative to norepinephrine, epinephrine (adrenaline) has a high affinity for α1-, β1-, and β2-adrenergic receptors. The β-effects are predominant at low doses, whereas the α1-effects are significant at higher doses. [2]. The potent β-action of epinephrine could compensate for the reflex decrease in maternal heart rate and cardiac output during SA for cesarean section [52].

#### Prevention of hypotension

A large drop in blood pressure during a cesarean section can endanger both the mother and fetus. Therefore, it is essential to take measures to ensure optimal circulatory stability during a cesarean delivery [46]. Indeed, fetal perfusion is dependent on uteroplacental blood flow, which is not autoregulated, making it directly dependent on the uterine perfusion pressure and inversely proportional to uterine vascular resistance [64]. There are several measures for preventing hypotension during SA for cesarean section that should be performed simultaneously. After administration of SA, crystalloid co-loading can begin with prophylactic administration of vasopressors [49]. The first option is phenylephrine infusion (syringe pump/infusion device) starting at 25–50 µg per minute after the administration of SA and adjusted for blood pressure and heart rate. Therefore, additional IV bolus therapy may be required. The second option is phenylephrine, which is administered as a bolus during the administration of SA [65]. A prefilled syringe is preferred due to reduced medication errors, less wastage, and patient safety [66–68]. An alternative option is an ephedrine bolus administered upon administration of the spinal anesthetic [68]. A maximum dose of 15 mg ephedrine can be used to avoid fetal acidemia. In women with preexisting hypertension or preeclampsia, caution is advised before initiating pharmacological measures to prevent hypotension. Non-pharmacological measures to prevent hypotension include tilting the table to 30° and manually shifting the uterus to the left [2].

#### Recognition of early hypotension

Three consecutive noninvasive blood pressure (NIBP) measurements are required to obtain the mean baseline NIBP (systolic pressure). At the same time, three simultaneous heart rate measurements are required to obtain the baseline heart rate values. The alarm threshold of the NIBP monitoring system is then set to 90% of the baseline NIBP value, with automatic measurement every 1 min or less, at least until delivery [2]. Frequent and continuous communication with the parturient until delivery may allow early recognition of hypotension. Nausea and vomiting after SA are among the earliest clinical signs of hypotension, as is subjective discomfort of the parturient [41]. Sudden tachycardia immediately after SA may precede the recognition of hypotension [2].

#### Vasopressor agent choices for hypotension management

SA-associated hypotension in cesarean delivery should be primarily managed with vasopressors rather than fluids [46]. Maternal and fetal outcomes are better when vasopressors are administered prophylactically rather than reactively to treat hypotension. Availability, cost, and benefit must be considered when selecting vasopressors for the treatment and prevention of hypotension (phenylephrine, ephedrine, norepinephrine, epinephrine). Phenylephrine has important properties for the treatment of spinal hypotension: (1) it is an α-adrenergic agonist that acts directly on the decrease in systemic vascular resistance after SA [38], (2) it has a faster onset of action than ephedrine [12], (3) ephedrine is associated with a five-fold increased risk of fetal acidosis [69], and (4) ephedrine is more likely to cross the placenta and increase lactate, glucose, and catecholamine concentrations in the fetal circulation than is phenylephrine [61]. Therefore, phenylephrine is the first-line vasopressor agent of choice for hypotension due to SA for cesarean section in parturients with normal heart rates. Given the maternal response to SA (decreased systemic vascular resistance), the use of ephedrine is not appropriate from the physiological point of view because of its beta-adrenergic agonist properties [26]. For bradycardia, however, ephedrine is the drug of choice. Adrenaline (epinephrine and norepinephrine) is the preferred choice for bradycardia with hypotension that does not respond to ephedrine treatment. If hypotension persists despite aggressive interventions, causes other than SA should be considered.

#### Vasopressor agent form for hypotension management

In a randomized double-blind clinical trial evaluating hemodynamic changes, administration of phenylephrine as an infusion did not provide clinical benefits compared with bolus treatment. The bolus regimen maintained maternal arterial blood pressure near baseline in the first minutes after SA, whereas the infusion treatment required a higher total dose of phenylephrine to have the same effect during the period prior to delivery [70]. In a recent study comparing bolus doses of 50 μg phenylephrine with a fixed infusion rate of 50 μg/min of the same prophylactically administered drug, the infusion group showed better control of blood pressure than the bolus group, although the neonatal outcome was similar in both groups [71]. Another study compared variable infusion, fixed on-and-off infusion, and intermittent boluses of phenylephrine for the prophylaxis of maternal hypotension during cesarean delivery. It concluded that the fixed on-and-off infusion regimen of phenylephrine provided the same cardiovascular profile as variable infusion and avoided unnecessary medical interventions and calculations [72].

Prophylactic administration of ephedrine via the intramuscular route is highly debatable because the systemic absorption and peak effect are difficult to predict, which can lead to rebound hypertension [73]. Although large doses are used, the IV route is more controllable and effective [62]. Studies using a prophylactic bolus or infusions of IV ephedrine have shown their efficacy in preventing episodes of hypotension [74–76]. A prophylactic bolus of 12 mg ephedrine administered intravenously at the time of intrathecal block and rescue boluses have been shown to result in a lower incidence of hypotension after SA during elective cesarean section than IV rescue boluses alone [76].

Studies on the use of norepinephrine during SA for cesarean delivery have reported delivery by computer-guided infusion, fixed-rate infusion, and intermittent boluses [18–20]. Manually controlled infusion of norepinephrine in the range of 0–5 µg/min was proven to be effective in reducing the incidence of hypotension and provided more stable blood pressure control than for a control group receiving rescue boluses of 5 µg norepinephrine to treat hypotension when it occurred [21]. Recently, low-dose epinephrine infusion was also shown to prevent maternal hypotension and uteroplacental perfusion during SA cesarean delivery [52].

#### Vasopressor agent administration routes

Vasopressors have been delivered via a central venous catheter (CVC) due to potential extravasation concerns and subsequent tissue necrosis [77]. However, the use of vasopressors via peripheral IV catheters (PiVCs) rather than CVC has been growing [77].

A systematic review of the safety of vasopressor drug administration via PiVCs concluded that extravasation is rare and unlikely to lead to major complications [78]. All vasopressor agents can be administered through large peripheral IV or CVC lines, based on these current data. Epinephrine and norepinephrine should be administered via a CVC line. However, in urgent cases, these agents may be administered temporarily via a peripheral IV line. Norepinephrine should be diluted in water with 5% dextrose (D5W) [79].

### Prompt hypotension management

#### Non-pharmacological measures

The debate is still ongoing on whether the patient’s height influences the block level in SA. Several studies have reported no statistical correlation between block level and height [80–82]. Huang et al. [83] showed that the dose of local anesthetic was not adjusted according to height in many studies. This is an important point for the population of SEA, where most parturients are small. The block level was related to the length of the vertebral column [84]. Although height accounted for only 10.6% of the variation in vertebral column length, there was a statistically significant correlation between these two parameters [82]. Thus, block level should be chosen based on height, as supported by two studies [85, 86]. Huang et al. showed that the block level was highly dependent on the height of the parturient when using a low dose of bupivacaine [83]. A lower dose of local anesthetic results in less blockade and reduces the incidence of hypotension at SA; however, it may cause incomplete analgesia and muscle relaxation [87].

A recent prospective observational study showed that there is a 3.26-fold higher risk of hypotension in the supine position before SA than in the full lateral position before SA [88]. Previous studies demonstrated that the incidence of hypotension was low if the parturient was in a fully left lateral position until the start of surgery [89, 90]. Furthermore, transferring a full-term parturient from the left lateral position to the left tilt position prevented aortocaval compression better than transferring them from the supine position to the left tilt position [91].

Maternal hyperoxia increases the fetal partial pressure of oxygen and improves the acid–base status [92]; supplemental oxygen should be administered via a nasal cannula or face mask while conducting an SA. Because an adequate sensory block for surgery should cover the fourth thoracic dermatome, there is a concomitant worsening of pulmonary function. Significant decreases in peak expiratory flow, forced vital capacity, forced expiratory volume in one second, and forced expiratory flow in the midrange of forced vital capacity were demonstrated after SA in the supine position [93]. These conditions and SA-induced hypotension can cause fetal acidemia (77). Therefore, supplemental oxygen in an elective cesarean section is indicated in clinical practice for the well-being of the fetus.

The second peripheral IV line or CVC was recommended, one for rapid fluid exchange and the other for drug administration, including vasopressors and atropine. In addition to standard monitors, an arterial line may be considered in high-risk patients for hemodynamic measurements, including pulse contour and cardiac output [68]. A trained nurse or doctor should be in the theater for help and advice.

Another aspect of treatment is the maintenance of normothermia. It is estimated that perioperative hypothermia occurs in > 60% of patients who receive SA for cesarean delivery [94–96]. In these patients it significantly impairs thermal autoregulation by inhibiting vasomotor responses and tremors, even beyond the level of sensory block, causing thermal redistribution of heat from the core to peripheral tissues [97, 98]. In the absence of strategies to preserve normothermia, patients become hypothermic in the first 30–40 min of surgery and remain hypothermic postoperatively [99]. Hypothermia increases the risk of cardiovascular events, such as arrhythmias, myocardial ischemia, coagulopathy, greater blood loss, requiring transfusions, wound infections that show delayed healing due to decreased antibody and cell-mediated immune responses, and oxygen availability in peripheral wound tissues. In addition, changes in pH alter the kinetics and effects of various anesthetics and paralyzing agents, increase thermal discomfort, and are associated with delayed postanesthetic recovery [94, 100].

#### Pharmacological measures

Fluid loading is another aspect of the antihypotensive strategy. It can counteract the relative hypovolemia due to venodilation and help maintain hemodynamic stability by increasing venous return [101]. Despite the effectiveness of phenylephrine, a significantly higher frequency of hypotension has been observed in the absence of fluid administration [36]. In addition, the CAESAR study showed that a mixed infusion of hydroxyethyl starch (HES) and Ringer’s lactate more effectively reduces maternal hypotension than Ringer’s lactate infusion alone when combined with IV phenylephrine boluses. In addition, the reduction in the incidence of severe symptomatic hypotension or both was even greater [102]. Another study revealed that the co-load strategy is superior to preloading for preventing maternal hypotension [103]. A survey showed that many obstetric anesthetists favor fluid therapy in their clinical practice [104]. However, crystalloids are required in large volumes(> 15 ml/kg) to decrease the incidence of hypotension [105]. These large volumes result in adverse effects, such as increased central venous pressure [106], blood dilution, leading to a decrease in oxygen transport capacity [107], and the release of atrial natriuretic peptide, initiating diuresis, thus reducing the effect of volume load on blood pressure [108].

The first-line treatment for hypotension is early intervention with phenylephrine if the heart rate is maintained during SA. In one study, phenylephrine infusions (25–50 µg/min) maintained baseline maternal hemodynamics and were used in conjunction with SA. Vigilant use of phenylephrine bolus infusions (50–100 µg) targeting the maternal heart rate as a surrogate for cardiac output is also effective, especially in resource-limited settings. Alternatively, phenylephrine infusions (< 25 µg/min) with boluses (50–100 µg) can be used [65].

An IV 5–15 mg bolus of ephedrine can be used as a rescue vasopressor for bradycardia. Its clinical action is primarily due to the indirect release of norepinephrine from postganglionic nerve endings. The drug shows a delayed onset of action and a longer duration of action of approximately 60 min [109]. Moreover, the cumulative dose before delivery should not exceed 15 mg to avoid fetal acidosis.

Epinephrine or norepinephrine may be administered for persistent hypotension. However, the use of such a potent agent in non-intensive care settings, such as the labor ward, has raised concerns [2]. At a dose of 0.1 μg/kg/min, epinephrine infusion was more effective than phenylephrine at maintaining blood pressure close to baseline during SA, with a lower decrease in maternal heart rate and cardiac output [52]. The use of norepinephrine to prevent and treat hypotension during SA is new, and data in the literature are scarce. Continuous computerized infusion of norepinephrine (5 μg/ml) and phenylephrine (100 μg/ml) to prevent hypotension during SA have been studied. Norepinephrine was more effective than phenylephrine in maintaining blood pressure, as well as higher cardiac output and heart rate [18]. Intermittent IV norepinephrine boluses to prevent SA-induced hypotension during elective cesarean delivery may also be considered. A recent study suggested a 90% effective dose (ED90) of 6 µg for this situation, which was not associated with adverse outcomes [20].

The efficacy of anticholinergic agents, such as for prophylaxis for post-spinal hypotension in the non-obstetric field, has been demonstrated [110]. Nevertheless, there is insufficient data to warrant its use in obstetric practice. However, a high level of SA blocks the cardiac sympathetic nerve and causes the heart rate to decrease and the force of contraction to disappear. Therefore, simply increasing vasoconstriction might not effectively compensate for cardiac output. Atropine blocks the vagus nerve and drives up the heart rate [111].

Some studies suggest that ondansetron, a 5-hydroxytryptamine subtype 3 (5-HT3) receptor antagonist generally used for the prophylaxis and treatment of nausea and vomiting, may also reduce the hemodynamic changes induced by SA [112, 113]. Arterial hypotension and bradycardia can be caused by sympathetic nerve blockade and the Bezold–Jarisch reflex, mediated by peripheral serotonin receptors (5-HT3 type). Therefore, IV administration of ondansetron, to block type 3 serotonin receptors, could reduce SA-induced hypotension and bradycardia. Accordingly, a meta-analysis provided evidence that 5-HT3 antagonists are effective in reducing the incidence of hypotension and bradycardia. The effects were moderate and significant solely in the subgroup of patients undergoing cesarean delivery [114].

#### Prediction of hypotension

The prediction of hypotension is based on the anesthesiologist’s preoperative assessment, especially in parturients with coexisting pathologies or risk factors. However, in certain cases, spinal hypotension can be predicted by simple parameters, such as age, preoperative heart rate, and preoperative MAP (Kinsella et al. [2]).

#### Prefilled syringes

The purchase of prefilled syringes with frequently prepared anesthetics has been suggested as a cost-saving measure [66]. Numerous studies have shown that avoidable waste is greatly reduced for drugs available in prefilled syringes (phenylephrine and atropine) relative to drugs of the same class available in single-dose vials (ephedrine, glycopyrrolate) [66, 115–117]. In a study at an academic medical center, Atcheson compared the avoidable waste generated by phenylephrine and ephedrine to gain insight into the economic impact of using prefilled syringes [67]. During the study period, phenylephrine was available almost exclusively in prefilled syringes and was stored unopened but kept on hand in 76.6% of cases; it was wasted in only 2.6% of cases. Ephedrine was prepared for a similar proportion of cases (80.3%), but because it was compounded (filled) by the anesthesiologist in the operating room, ephedrine was wasted in three of five cases in which it was prepared. In addition, the difference in waste per case could have environmental implications [67]. Similar conclusions were drawn in another university obstetrics department, in which prefilled syringes significantly reduced ephedrine wastage, thus minimizing costs in obstetric anesthesia [118]. Prefilled syringes have advantages over standard syringes. They allow convenient and rapid administration of vasopressors during an emergency, saving time and lives. Medications stored in prefilled syringes remain sterile for 2–3 years. Prefilled syringes also improve the safety and accuracy of medication administration by leading to fewer dosing and medication errors [10, 11, 68, 119, 120].

### Current practice in the management of hypotension with vasopressors during cesarean section under SA: a Southeast Asia survey

The survey containing 17 statements was distributed as an anonymous Delphi e-survey to peers in the Philippines, Thailand, and Vietnam. The participants were asked to provide their input on the choice of vasopressors for managing hypotension among parturients undergoing cesarean section under SA. An agreement was reached if at least 75% of participants scored the statement as “strongly agree” or “agree” on a four-grade rating scale. For those statements for which no consensus was reached, the statements were revised based on the voters’ comments, followed by a second round of voting. If no agreement was reached after two rounds of voting, the statement was excluded.

A total of 183 completed questionnaires were analyzed from the Philippines (69.4%, *N* = 127), Vietnam (15.8%, *N* = 29), and Thailand (14.7%, *N* = 27). The vast majority of respondents agreed or strongly agreed with the following statements.

1. Hypotension during SA for cesarean delivery is common and must be recognized and treated immediately.
2. Prevention of spinal-induced hypotension is an important strategy to improve maternal and neonatal outcomes in cesarean deliveries. Fetal perfusion depends on uteroplacental blood flow, which lacks autoregulation so that it is directly dependent on uterine perfusion pressure and inversely proportional to uterine vascular resistance.
3. The duration of hypotension may be more important than its severity.
4. Systolic arterial pressure (SAP) must be maintained at ≥ 90% of an accurately measured baseline until delivery of the newborn to reduce the frequency and duration of episodes of significant hypotension to < 80% of baseline. SAP < 80% should be treated promptly, usually with a bolus injection of vasopressors. An SBP of > 90 mmHg was acceptable. In preeclampsia or preexisting hypertension, the target blood pressure should be adjusted individually.
5. Bradycardia at SA for cesarean delivery may result in low cardiac output and must be treated promptly with atropine or anticholinergics.
6. To prevent hypotension during SA for cesarean delivery, concurrent measures must be taken.
7. Prespinal measures to prevent hypotension in parturients after SA include leg compression devices, manual left uterine displacement, and administration of 5HT3 antagonists (e.g., ondansetron).
8. SA hypotension during cesarean delivery should be treated primarily with vasopressors complemented by fluid administration. Maternal and fetal outcomes are better when vasopressors are administered prophylactically rather than reactively to treat hypotension.
9. After successful SA, crystalloid co-loading and prophylactic administration of vasopressors should be started.
  - First choice: Phenylephrine administered:
    i. 1) Infusion (syringe pump/infusion device) began after administration of spinal anesthetic at 25–50 µg/min titrated to blood pressure and heart rate. Therefore, additional IV boluses may be required.
      OR
    ii. 2) As a bolus (ideally, use a prefilled syringe) after administration of SA without bradycardia. For immediate treatment of hypotension, an IV bolus of phenylephrine has a more rapid onset of action than an infusion.
  - Second choice: Ephedrine bolus administered upon administration of SA.
  - Alternative choice considering availability: Noradrenaline (Norepinephrine) as an infusion/bolus in resource-limited areas (via central line or temporarily via a wide-bore peripheral line).
10. Crystalloid co-loading starts immediately before or at the start of SA.
11. Considering the availability, patient safety, cost, and benefits when choosing vasopressors for hypotension prevention and management (phenylephrine, ephedrine, norepinephrine, and epinephrine).
  - Phenylephrine is the first choice vasopressor agent for hypotension from SA for cesarean delivery in parturients with a normal heart rate.
  - Ephedrine is a vasopressor of choice in the presence of bradycardia.
  - Both epinephrine and norepinephrine are alternatives to correct hypotension and bradycardia unresponsive to ephedrine.
12. When hypotension persists despite aggressive intervention, causes other than sympathetic blockade should be considered.
13. Whether phenylephrine is administered as an infusion or bolus depends on the available resources at the institution, the cost, and the best practices agreed upon locally.
14. Single-dilution techniques using phenylephrine prefilled syringes or both should be considered and are preferable for patient safety.
15. Emergency cesarean delivery requires careful assessment of volume status with consideration of potential losses, including hemorrhage, vomiting, and prolonged labor. Significant hypovolemia is a contraindication for SA. Sympathectomy after SA can result in a potentially fatal reduction in venous return and cardiac preload.
16. Women with preeclampsia develop less hypotension than healthy women after SA. An abrupt drop in blood pressure is undesirable because it may result in decreased uteroplacental blood flow. A prophylactic vasopressor infusion may not be necessary; however, if used, it should be started at a lower rate than in healthy women.
17. Women with heart disease should be evaluated individually. Some conditions are best treated with phenylephrine (an arterial constrictor with no positive inotropic effects), whereas others respond best to ephedrine (which has positive inotropic and chronotropic effects).

### Regulatory considerations

The summary of healthcare systems in the Philippines, Thailand, and Vietnam; and the main challenges are summarized as follows (Table 2).

**Table 2:** Summary and main challenges of healthcare systems in the Philippines, Thailand, and Vietnam

Philippines	Thailand	Vietnam
Phenylephrine is not listed in the Philippine National Drug Formulary (PNDF) but is available in the Philippine market on the rationale of “compassionate use.” The Philippine National Health Insurance (Philhealth) *reimburses only PNDF-listed medications used by patients.* Non-PNDF medications are paid out-of-pocket by patients in public hospitals. Therefore, it remains an additional financial burden for the patient. The higher cost is an issue compared to that of ephedrine	Phenylephrine is new and recently included in Thailand's Diagnostic Related Group. However, anesthesiologists are concerned about the wasted cost of the unused drug when prepared in anticipation of use. The prefilled syringe can be cost-saving. Norepinephrine and epinephrine are used in some hospitals when phenylephrine is not available	Phenylephrine is registered but is limited to specialty hospitals. This deprives smaller community hospitals of medications like phenylephrine. On the other hand, ephedrine is cheap and widely available. Recently, Vietnamese health care insurance has inserted phenylephrine into the drug list. It means that insurers pay for this drug

### Checklist for hypotension management

*Prophylactic vasopressor should be administered straight after SA.*

*Alpha-agonists are the most physiological, and phenylephrine is currently recommended.*

*Tilt the parturient 30° laterally and co-load the vasopressor with crystalloid.*

*Aim to maintain SBP* > *90% baseline and avoid significant hypotension* < *80% baseline.*

*When using variable rate infusion, start at 25–50 µg. min-1 phenylephrine, plus boluses PRN.*

*Administer phenylephrine bolus (prefilled syringe preferred) at 50–100 µg or ephedrine bolus at 5–15 mg.*

*Heart rate is a surrogate for cardiac output.*

*Use low doses of ephedrine for hypotension with low heart rate and anticholinergic for bradycardia.*

*Smart pumps provide greater stability.*

*If needed in case of preeclampsia, start vasopressors with lower doses.*

*Individualize the decision in the presence of cardiac conditions.*

*Refractory hypotension is individualized according to coexistent conditions contributing to shock, e.g., heart failure, arrhythmias, organ ischemia, or agent availability.*

*- Norepinephrine: agent of first choice*

*- Dobutamine for cardiogenic shock*

*- Epinephrine (adrenaline) for anaphylaxis*

*- Phenylephrine if tachyarrhythmia*

*Avoid vasopressin since it increases uterine contractions.*

*Avoid dopamine since it is associated with an increased risk of death (surviving sepsis campaign) in septic shock.*

### Recommendations for the management of hypotension after SA for cesarean section in limited-resource environments

#### Choice of the vasopressor

After careful assessment of volume status and the exclusion of hypovolemia:

- Phenylephrine is the vasopressor of choice, if available.
- Infusion options if no syringe driver is available:
- No infusion: A bolus of 50*–*100 µg phenylephrine is required. Start treatment when heart rate increases, SBP decreases to 90% baseline, or both.
- Infusion: 500 µg phenylephrine is added to the first liter of Ringer’s lactate and administered rapidly after SA. Administration for 10*–*20 min is approximately equivalent to an infusion of 25*–*50 µg/min and can be titrated according to heart rate and blood pressure responses.

Alternative options:

- Ephedrine (50 mg) added to 10 ml 0.9% saline in a 10-ml syringe (5 mg/ml): Bolus dose of 10 mg.
- Adrenaline (0.5 mg) added to 100 ml 0.9% saline (5 µg/ml): Bolus dose of 10 µg.

#### Monitoring

The following values should be recorded (repeat the baseline measurements if they are outside the normal range):

- 1.Baseline SAP
- 2.90% baseline SAP
- 3.80% baseline SAP

#### At the end of the SA injection

Commence vasopressor infusion at a predetermined starting rate.

Set NIBP measurements to 1 min cycles or perform manual BP measurements every 1 min until SBP is stable or the baby is delivered.

Start IV crystalloid co-loading:15 ml/kg (titrated accordingly).

Treat hypotension early and aggressively. Aim to keep the SAP ≥ 90% of baseline SAP and heart rate ≤ 120% of baseline.

Be cautious for patients with heart failure, kidney disease, or congestion.

### Management techniques scheme (Fig. 2)

**Fig. 2:**
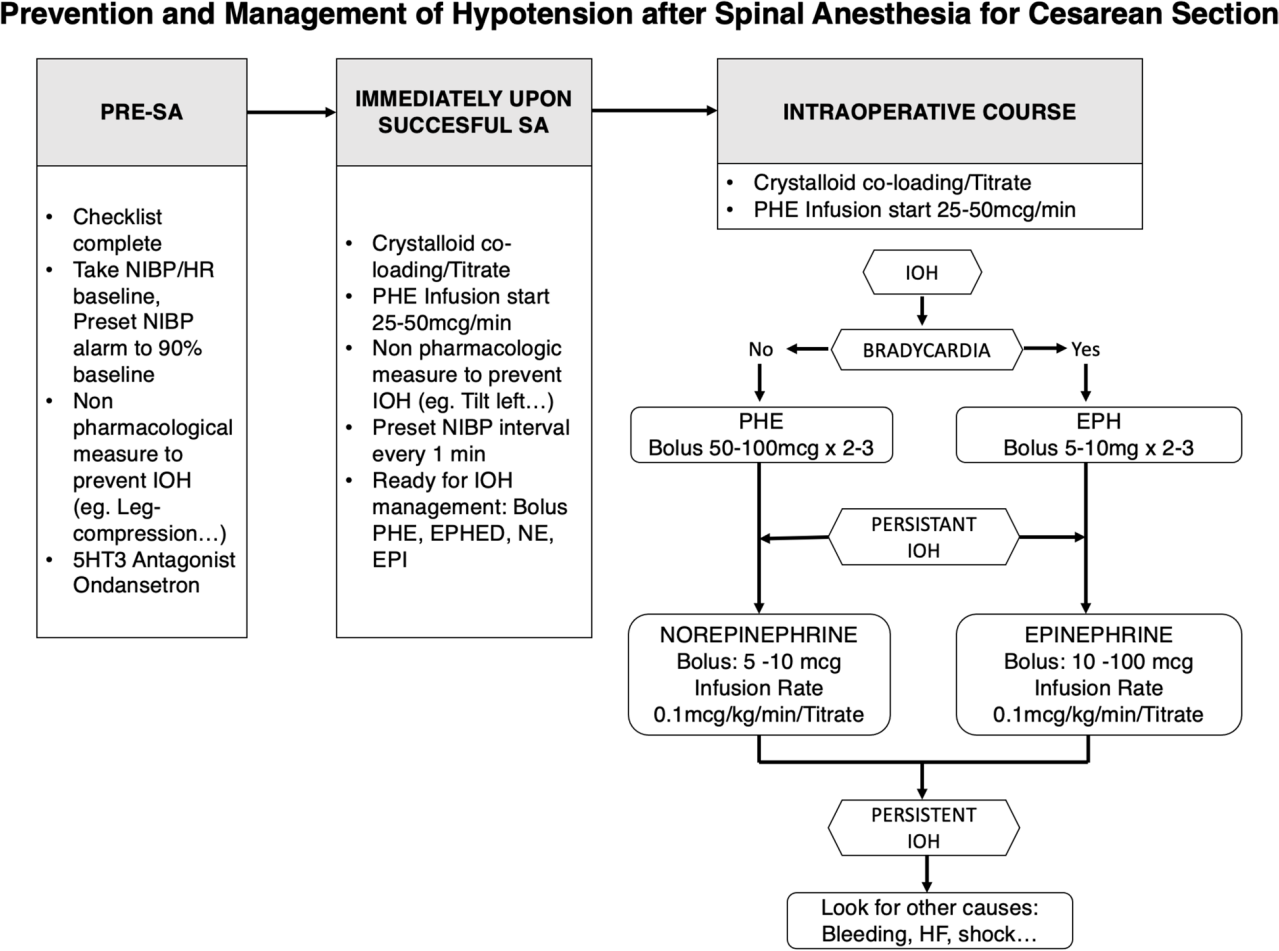
Management techniques scheme

## Data Availability

The data of the present expert consensus are available at reasonable request from the corresponding author.
